# Supplementary material for: Use of three points to determine the accuracy of guided implantation
Source: PLoS One. 2019 Dec 3;14(12):e0225823. doi: 10.1371/journal.pone.0225823 (PMC6890217; doi:10.1371/journal.pone.0225823)
Supplement: S1 File — Error analysis and calculation in the TriP image registration method Calculation and correction of cover Screw Height (HS) and other indicators Calculation of accuracy indicators CRD and ARD Traditional image registration method Design and fabrication of VDING guide template Accuracy evaluation for the VDING template Deduction of influence of the distance on system error Patient inclusion criteria and information. (DOCX) [file pone.0225823.s001.docx]

Use of three points to determine the accuracy of guided implantation

Ye Liang^1¶^, ShanShan Yuan^3¶^, JingJing Huan^1^, HuiXin Wang^1^, YiYi Zhang^1^, ChangYun Fang^1&*^, Jia-Da Li^2&*^

^1^ Department of Stomatology, Xiangya Hospital of Central South University, Changsha, Hunan Province, China

^2^ School of Life Sciences, Central South University, Changsha, Hunan Province, China

^3^ Affiliated Stomatology Hospital of Guangzhou Medical University

* Corresponding author

E-mail:

fcy@kuye.cn (CYF)

lijiada@sklmg.edu.cn (JDL)

^¶^Both authors contributed equally to this study as first authors.

^&^Professor ChangYun Fang and Professor Jia-Da Li contributed equally to this study.

Supporting Information

Registration of the planned image and the real implant image

The points in the planned image are in coordinate system P, and the points in the real implant image after cone beam computed tomography (CBCT) are in coordinate system R.

The corresponding landmark points $L_{1},L_{2},L_{3}$ are chosen in the 2 images. The points in the planned implant image (coordinate system P) are $P_{L1},P_{L2},P_{L3}$. The corresponding points in the real implant image (coordinate system R) are $R_{L1},R_{L2},R_{L3}$. The coronal and apical endpoints of the implant are $I_{C}$ and $I_{A}$, respectively. Therefore, the coronal and apical endpoints are respectively $P_{\mathrm{IC}}$ and $P_{\mathrm{IA}}$ in the planned implant image and $R_{\mathrm{IC}}$ and $R_{\mathrm{IA}}$ in the real implant image.

With the geometric transformation method described in 2.1.2, the coordinate system P' can be established with the landmarks $P_{L1},P_{L2},P_{L3}$. The obtained coordinates of points $N_{\mathrm{PIC}}$ and $N_{\mathrm{PIA}}$ correspond to points $P_{\mathrm{IC}}$ and $P_{\mathrm{IA}}$, respectively, in coordinate system P. The coordinate system R’ can be established with the landmarks $R_{L1},R_{L2},R_{L3}$. The obtained coordinates of points $N_{\mathrm{RIC}}$ and $N_{\mathrm{RIA}}$ correspond to points $R_{\mathrm{IC}}$ and $R_{\mathrm{IA}}$, respectively, in coordinate system R (S1 Fig).


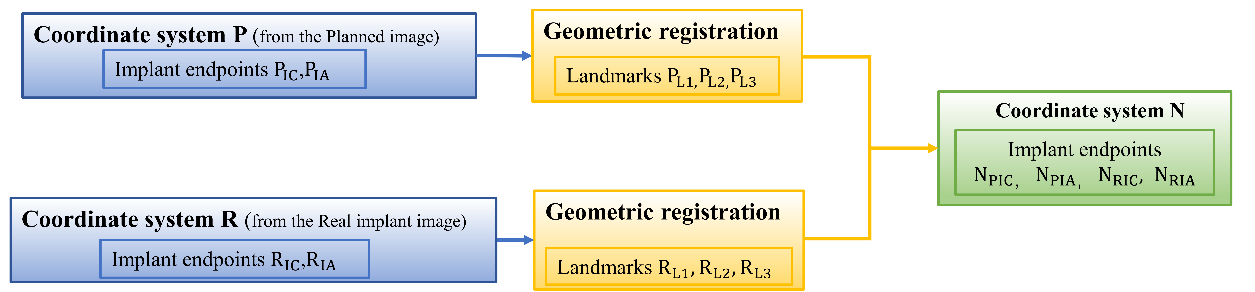


**S1 Fig. Spatial Transformation of Points.**

The three pairs of landmark points, $P_{L1}\sim R_{L1},P_{L2}\sim R_{L2},P_{L3}\sim R_{L3}$, chosen from the planned image and the real implant image are counterparts of the same anatomical features. Therefore, under the assumption of a negligible selection error, the rigid spatial data indicating the location of anatomical landmark points on the skeleton will be completely consistent in coordinate system P' and coordinate system R'. Therefore, coordinate system P' and coordinate system R' can be referred to as the same coordinate system - coordinate system N (N for new). Hereto, both the planned image and the real implant image are projected onto a common reference space. The planned and real implants can be compared in coordinate system N. From the coordinates of the points $N_{\mathrm{PIC}}$, $N_{\mathrm{PIA}}$, $N_{\mathrm{RIC}}$, and $N_{\mathrm{RIA}}$, the indicators of implant deviation can be calculated (S2 Fig).


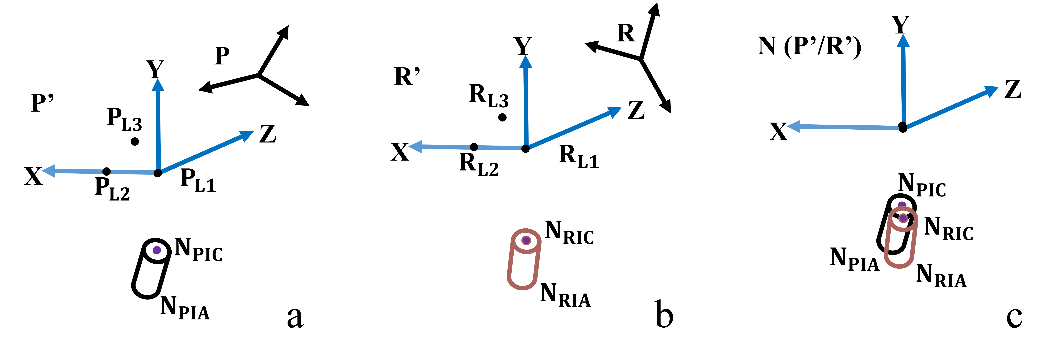


**S2 Fig.** **Diagram Showing Coordinate System P' and Coordinate System R' as the Same New Coordinate System.** (A) In the coordinate system P', the coronal and apical endpoints of the planned implant are N_PIC_ and N_PIA_, respectively. (B) In coordinate system R', the coronal and apical endpoints of the real implant are N_RIC_ and N_RIA_, respectively. (C) Coordinate system P' and coordinate system R' are regarded as the same coordinate system – N. The deviation can be calculated with the coordinates obtained for the 4 endpoints of the implant.

Error analysis and calculation in the TriP image registration method

The selection of landmarks and target points will have errors associated with the image resolution and manual operation. The existence of errors affects the distance between the landmarks in coordinate systems P and R, which are located in the same space; this distance should be greater than 0 in the transformed space – the coordinate system N, and deviations reflect the system error of the TriP method.

When we analyze target points, the results will also be affected by system errors. A higher image resolution will correspond to a more precise selection of points, a smaller system error, and greater reliability in the calculation of deviations.

Due to the different original images used in different studies and the differences in the methods of different operators, the system errors will be different in different cases. To evaluate the system error of each case objectively, it is necessary to set up a method of calculating system errors to analyze the guide template accuracy more correctly and accurately.

In the TriP method described above, we define the system error according to the following methods:

STEP 1: arrange $E_{n}$ of each pair of landmark points in increasing order according to the value calculated by the method defined in 2.1.4. Then, we obtain the sequence {$E_{n}$}.

{$E_{n}$}=$E_{1},E_{2},E_{3},E_{4},\ldots,E_{n}$

STEP 2: select points corresponding to $E_{1},E_{2},E_{3}$ and term them $L_{1},L_{2},L_{3}$, respectively. Use the method explained in 2.1.3 with these 3 pairs of points to build the coordinate system N.

STEP 3: select the pair $P_{\mathrm{Lm}}$ and $R_{\mathrm{Lm}}$ that corresponds to $E_{4}$ as a transient target point. Use the method in 2.1.3 to perform the geometric transformation and obtain the coordinates of $N_{\mathrm{PLm}}{, N}_{\mathrm{RLm}}$.

STEP 4: calculate the length of the segment $N_{\mathrm{PLm}}N_{\mathrm{RLm}}$. This length is considered the system error of TriP.

The system error will reflect the point selection error and the geometric transformation error.

Calculation and correction of cover screw height (HS) and other indicators

Use the method described in 2.1.3 to opt for the best 3 pairs of landmarks to perform geometric transformation. Then, use the method described in 2.1.2 to obtain the coordinates of the coronal endpoint N_PIC, the apical endpoint N_PIA of the planned implant, the coronal endpoint N_RIC and the apical endpoint N_RIA of the real implant. Since it is more conducive to infection prevention to reexamine the CBCT images after implant surgery, rather than during the surgery, there is usually a cover screw or healing base detectable on the real implant images. As a result, the measured implant length $N_{\mathrm{RIC}}N_{\mathrm{RIA}}$ is larger than the planned implant length $N_{\mathrm{PIC}}N_{\mathrm{PIA}}$.

To correct the disparity in height, it is considered that the implant base position is not affected by the upper structure, and we must correct for the additional height. It is considered that the implant base position is not affected by the upper structure. Therefore, as shown in Fig 1g, we can construct a point $N_{\mathrm{RIC}}'$ on the line segment $N_{\mathrm{RIC}}N_{\mathrm{RIA}}$ to create ${N_{\mathrm{RIC}}{'N}_{\mathrm{RIA}}=N}_{\mathrm{PIC}}N_{\mathrm{PIA}}$.

The $N_{\mathrm{RIC}}N_{\mathrm{RIC}}'$ that we obtain is the height of the cover screws or healing base. The following indicators for implant endpoints were calculated using $N_{\mathrm{RIC}}'$.

Calculation of accuracy indicators CRD and ARD

CRD and ARD are calculated after correcting for the positions of the crown and apical endpoints along the long axis of the real implant. After correction, the value of indicators will not be affected by the subjective adjustment of the implant height during the manual implant insertion stage.

The specific calculation methods are as follows.

STEP 1: Draw a vertical line over the $N_{\mathrm{PIC}}$ perpendicular to $N_{\mathrm{PIC}}N_{\mathrm{PIA}}$, intersecting $N_{\mathrm{RIC}}'N_{\mathrm{RIA}}$ at point $N_{\mathrm{RIC}}''$.

STEP 2: Construct a point $N_{\mathrm{RIA}}''$ on the extension line of $N_{\mathrm{RIC}}'N_{\mathrm{RIA}}$ to create $N_{\mathrm{RIA}}N_{\mathrm{RIA}}''=N_{\mathrm{RIC}}'N_{\mathrm{RIC}}''$.

STEP 3: Define CRD as the length of $N_{\mathrm{PIC}}N_{\mathrm{RIC}}''$ to indicate the coronal deviation after correction of the coronal depth.

STEP 4: Define ARD as the length of $N_{\mathrm{PIA}}N_{\mathrm{RIA}}''$ to indicate the apical deviation after correction of the coronal depth.

Follow by obtaining the value of the CRD and ARD indicators (Fig 1h).

Traditional image registration method

CBCT images of postoperative patients were read, and image registration was performed with Mimics software, version 10.01 (Materialise n.v., Belgium), in Shanghai Ninth People’s Hospital.

According to the diameter and length of the implant, a cylinder was preliminarily generated, and the endpoints of it were carefully moved on the enlarged detail drawing to coincide with the real implant. In this way, the cylinder could represent the characteristic position of the actual implant. Then, the cylinder was further converted into a STL data model and saved as an MS file awaiting registration.

Subsequently, the CBCT images of preoperative patients are opened with MIMICS software, version 10.01. The coordinates of planned implant endpoints and the diameter of the implant are obtained from the E-3D Implant ×64 implant template design software ( software jointly developed by the center of Stomatology of Xiangya Hospital of Central South University and the Institute of Information Science and Engineering of Central South University). With these coordinates, an accurate cylinder is established to replace the planned implant.

Next, the registration function of MIMICS is used to import the saved real implant MCS files, followed by adjustment of the brightness, contrast and image size. Registration of the two images is performed to assess the targeted jaw in which the implant is positioned to obtain the maximum coincidence (S3B Fig), followed by derivation of the vertices of the two cylinders representing the planned implant and the real implant, respectively. The top and bottom center points of the planned implant and the real implant can be obtained by traditional registration methods (S3 Fig).


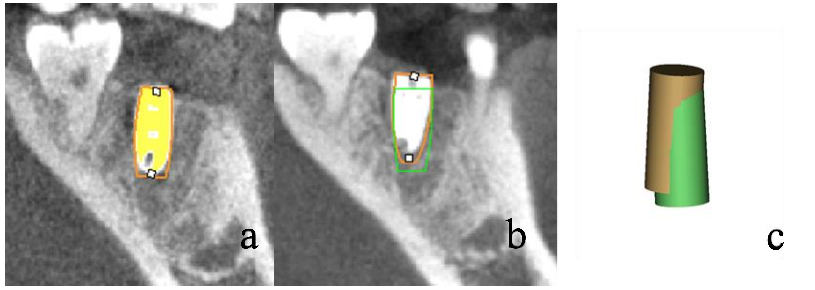


**S3 Fig. Steps of Traditional Image Registration.**

Design and fabrication of VDING guide template

Refer to the method of [1] for the following steps to make a VDING guide.

STEP 1: Personalized jaw data collection. A CBCT machine (KaVo Dental GmbH, Biberach, Germany; 0.25-mm slice interval; Imaging Sciences International 17-19 System) is used to scan the jaws of the patients. During the sampling process, the patient is instructed to bite on cotton balls to maintain a stable and slight mouth opening position. The scanning diameter and height are 16 cm and 13 cm, respectively.

STEP 2: Personalized teeth morphology acquisition. The morphology of the upper and lower dentition is obtained by alginate impression material and is further molded by plaster modeling. The data of the mandibular and maxillary gypsum models are collected by an optical scanning system and -3shape R2000 scanner software and are stored as STL files.

STEP 3: Data importation, registration and surgical simulation. The CBCT data and maxillary and mandibular STL files are imported into E-3D Dental master ×64. Registration is performed by automatic registration and manual adjustment (S4A Fig), followed by describing of the curvature of the dental arch (S4A Fig) and designing of the crown at an appropriate position (S4C Fig). The ideal position of the implant is virtually determined in accordance with the bone mass, important locations of anatomical structure, aesthetic requirements, gingival morphology and implant system characteristics (S4 Fig).


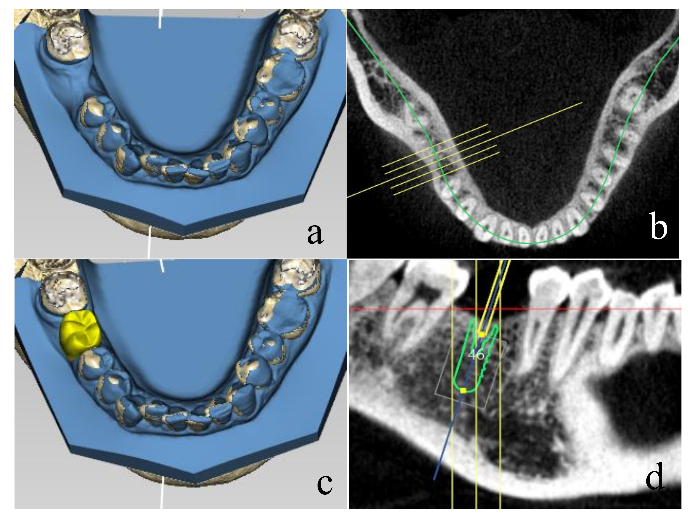


**S4 Fig. Locating the Position of the Planned Implant in the Software.** (A) Drawing of the arch curvature. (B) Aligning the CBCT data with maxillary and mandibular plaster model STL data. (C) Designing the crown position. (D) Virtually determining the implant position.

STEP 4: Design and manufacture of the VDING guide template. According to the planned implant position, placement direction of the guide template is set in E-3D Implant ×64 (S5A Fig), and the placement range of the guide template was delimited (S5B Fig). According to the length of the drill needle required for selected implants, a drill stop platform is designed that can define the maximum depth of the drill needle into the bone as planned. The guide sleeve is designed separately to match the different diameters of different levels of the drill needle. Two to four cylinders are designed parallel to the long axis of the implant as a visual, directional indicating device. The placement of cylinders should not hinder the movement of the drilling machine and placement of the guide template into the mouth. Finally, the VDING guide template is finished by placement of a diversion hole for water cooling on the guide template (as shown in S5C Fig). Through these processes, the corresponding VDING guide templates are finished according to the required drill needles, and the files are saved in STL format.


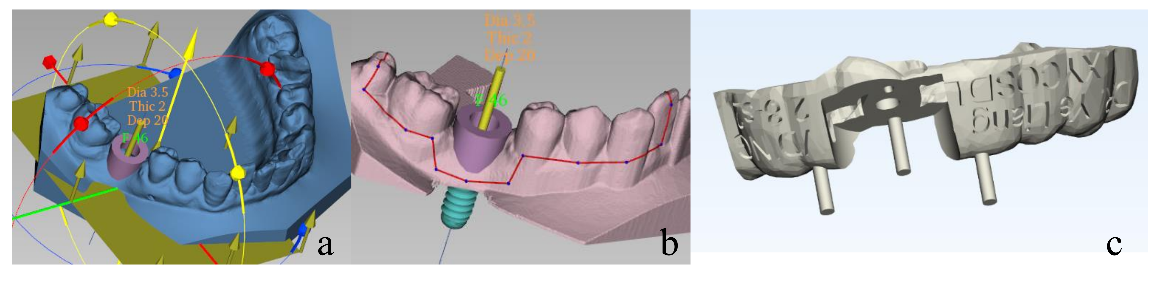


**S5 Fig. Design and Manufacture of Guide Template.** (A) Establish the placement direction of the guide template. (B) Delimit the placement range of the guide template. (C) Complete VDING guide template design.

STEP 5: Fabrication of the guide template: The STL file of the VDING guide template is imported into the Slice Setting Software (3-D Modeler Client Manager 5.9.7973.0) for typesetting and slicing. The XHD mode (accuracy of 16 micron) is used to submit the printing task to the 3D printer (3D Systems, ProJet3510 HD-plus). The surgical guide template is made of crystal material, which has been certified as a Class VI medical device in the United States, and it can be used in contact with human tissue in surgery. After printing, the standard process is executed, and the guide template is manufactured.

Accuracy evaluation for the VDING template

The maximal mouth opening varies among different races, genders and tooth sites, and the size of the opening ranges from 32 mm to 62 mm [2,3]. Although implantation can be carried out when the occlusal distance of the targeted site is slightly insufficient, it is difficult for the drill needle to fit into the guide sleeve because of the height of the guide sleeve after the digital guide template is applied [4]. The conventional solution is to use short drilling needles [5,6] (S6 Fig) or short guide sleeves [7,8] (S7 Fig).


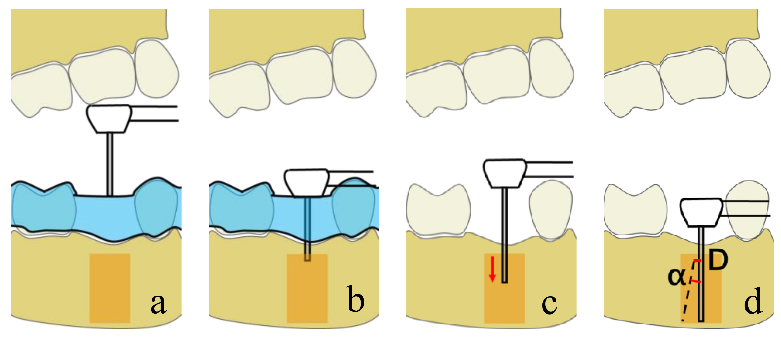


**S6 Fig. Guide Template Operated with a Short Drill Needle.** (A) Place the implant hand piece with a short drill needle and a guide template with a normal-height guide sleeve into the patient's mouth. (B) Use a short drill needle and a guide template with a normal-height guide sleeve to prepare the upper part of the implant socket. (C) Remove the guide template and drill down along the upper part of the bone socket. (D) Manually drilling down to the required depth will produce distance and angle deviations, decreasing the accuracy.


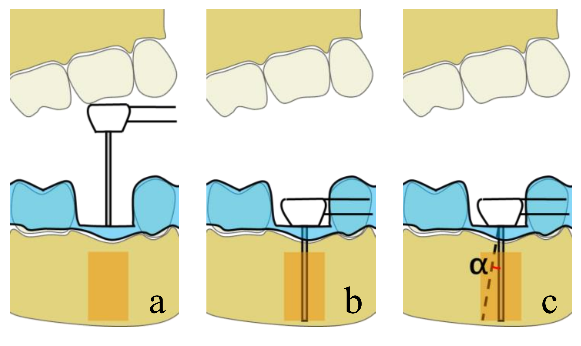


**S7 Fig. Guide Template with a Short Guide Sleeve** [1]**.** (A) A conventional drill needle and a short guide sleeve can be placed in the patient's mouth. (B) A template with a short guide sleeve is used to guide the drill needle downward. (C) The angle of the drill needle is likely to deviate during the movement, decreasing the accuracy.

When a short drill needle is used, the guide sleeve can maintain a higher height, but the drill needle cannot reach the planned depth. Therefore, the preparation of the upper part of the implant socket is similar to that of the conventional preparation. For the lower part of the socket, the guide template must be removed, and drilling is performed downward [5,6] along the upper part of the socket. Because of the manual drilling performed in the lower section, the accuracy of implantation will be reduced [6] (S6 Fig).

When a short guide sleeve is used, the conventional drill needle can be used and reach the target depth to complete the depth-controlled dental implant socket preparation. However, the angle control with a short guide sleeve is always inadequate [7]. Further, due to the inhomogeneous density of alveolar bone, the direction of the drill needle movement is prone to deviation [1] (S7 Fig).

To solve the above problems, a new type of implant guide template with a visual direction-indicating device (VDING) was designed, and we measured its precision in this study. A visual direction-indicating device is added to the traditional guide template to achieve functional enhancement in addition to direction control outside the guide sleeve (S5C Fig). In practice, it was found that the device could compensate for the shortcomings of insufficient angle limitation caused by insufficient height of the guide sleeve in cases where the implant site was too narrow for the template with a normal-height guide sleeve. During the implantation process, VDING enables doctors to observe the direction of implantation in real time, which is helpful for judging the position of the guide template and the direction of implantation over time (S8 Fig).


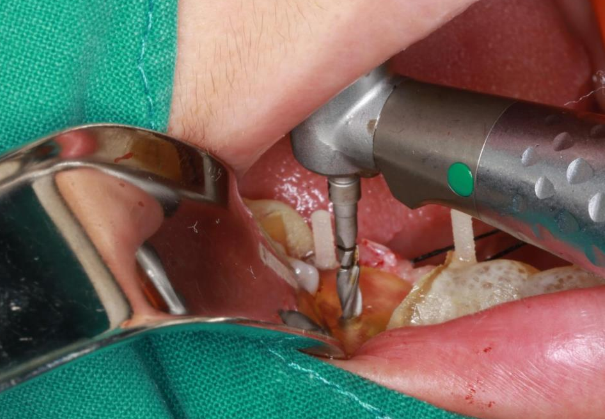


**S8 Fig. The VDING Template is Used During Implant Surgery.** The implant hand piece is controlled such that the drill needle is parallel to the visual direction-indicating device.

Using the VDING template for implantation, the angle deviation (AD), coronal global deviation (CGD), coronal vertical deviation (CVD), coronal lateral deviation (CLD), apical global deviation (AGD), apical vertical deviation (AVD), and apical lateral deviation (ALD) are 5.57±1.91 degrees, 1.17±0.50 mm, -0.76±0.85 mm, 0.49±0.33 mm, 1.74±0.51 mm, 0.82±0.85 mm, and 1.30±0.50 mm, respectively. After correction for coronal depth, the crown deviation (CRD) is 0.50±0.35 mm, and the apical deviation (ARD) is 1.37±0.49 mm.

Because VDINGs are especially suitable for patients with small mouth openings, these guides may play an important role in treating specific patients and improve accuracy when the guide sleeve of the template provides insufficient angular control.

However, comparison with the results in the literature indicates that long guide sleeves provide more mechanical orientation constraints and more directional control than visual directional sleeves, indicating that devices with long guide sleeves should be used at implant sites with wider interarch distances.

Deduction of influence of the distance on system error

To deduce whether the spacing between selected landmarks should be larger or smaller, points $P_{A}，P_{B}$ in the planned image and points $R_{A}，R_{B}$ in real implant image were selected.

Because of point selection error, $P_{A}，P_{B}$ do not coincide with each other in the actual anatomical structure of the patient, nor do $R_{A}，R_{B}$ (S9 Fig).


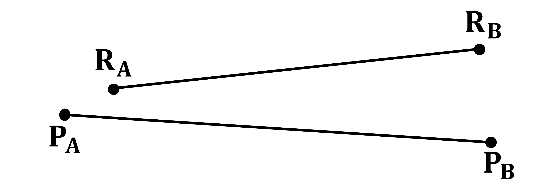


**S9 Fig. Selected Points do not Overlap in the Actual Anatomical Structure.**

By using the method in 2.1.3, the transformed points $P_{A}$ and $R_{A}$ will coincide, which means that the two images are aligned by translation of the pair of A points. Because this pair of points A is now regarded as error-free, a certain point-selecting error is introduced into the system. By using the method in 2.1.4, the pair of points A is the pair with the lowest error, so the error can be reduced only by a more precise selection.

The second step of the transformation will make the direction of $\vec{P_{A}P_{B}}$ consistent with that of $\vec{R_{A}R_{B}}$, which means that the two images will be rotated around the overlapped $P_{A}$ and $R_{A}$ so that $P_{A}$, $R_{A}$, $P_{B}$ and $R_{B}$ are collinear. In this process, the angle between 2 vectors is regarded as zero, resulting in an angular deviation between the two images (S10 Fig).


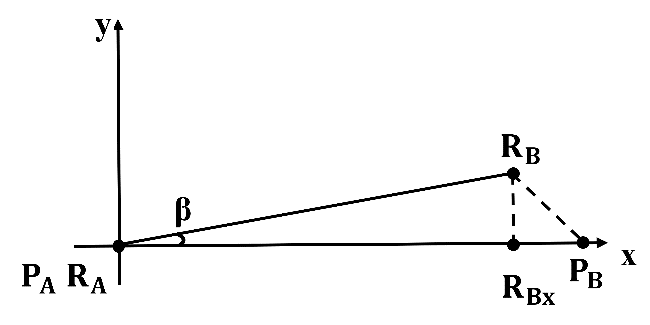


**S10 Fig. Angular Deviation in Registration.**

If the angle between $P_{A}P_{B}$ and $R_{A}R_{B}$ is β, decreasing β will help reduce the system error. If a line perpendicular to $P_{A}P_{B}$ is drawn through the point $R_{B}$, and the projected point is $R_{\mathrm{Bx}}$, then tanβ=$\frac{R_{B}R_{\mathrm{Bx}}}{P_{A}R_{\mathrm{Bx}}}$. The conventional error of one pair of points is less than 1 mm, and the distance between two different points is far greater than the error. Therefore, $R_{B}R_{\mathrm{Bx}}$ is regarded as a constant, $P_{A}R_{\mathrm{Bx}}\approx P_{A}P_{B}$. That is, a longer the distance between the two selected points will correspond to a smaller tanβ and a smaller β, considering the change rule of tanβ within 90 degrees. The three pairs of points are the same when establishing three-dimensional coordinates: a more scattered selection of landmark points can decrease the angular error.

Patient inclusion criteria and information

Inclusion criteria: All patients met the following conditions: age over 18 years old; dentition defect; no local or systemic contraindication for implant surgery; vertical dimension of the implantation-needed area greater than 30 mm when the mouth opening is at its maximum; sufficient bone at the implant site or sufficiency of a small-scale bone graft for implantation; willingness to sign an informed consent for the study. The details of these cases are given in S1 Table.

**S1 Table. Details of Selected Case.**

| Implant ID | Patient ID | Gender | Age (years) | Implant site (FDI) | Implant system | Implant length (mm) | Diameter (mm) |
| --- | --- | --- | --- | --- | --- | --- | --- |
| 1 | Patient 1 | Male | 20 | 46 | Zimmer | 10 | 4.7 |
| 2 | Patient 2 | Male | 39 | 26 | Zimmer | 10 | 4.7 |
| 3 | Patient 2 | Male | 39 | 47 | Zimmer | 8 | 4.7 |
| 4 | Patient 3 | Female | 44 | 11 | Zimmer | 13 | 3.7 |
| 5 | Patient 4 | Female | 56 | 47 | Zimmer | 8 | 4.7 |
| 6 | Patient 5 | Male | 30 | 15 | Zimmer | 10 | 4.1 |
| 7 | Patient 6 | Female | 23 | 26 | Zimmer | 8 | 4.7 |
| 8 | Patient 7 | Female | 43 | 36 | Zimmer | 11.5 | 4.1 |
| 9 | Patient 8 | Male | 43 | 32 | Zimmer | 13 | 3.7 |
| 10 | Patient 8 | Male | 43 | 42 | Zimmer | 13 | 3.7 |
| 11 | Patient 9 | Female | 35 | 46 | Zimmer | 10 | 4.7 |
| 12 | Patient 10 | Female | 28 | 46 | SLActive | 10 | 4.1 |
| 13 | Patient 11 | Female | 61 | 23 | Zimmer | 13 | 3.7 |
| 14 | Patient 12 | Female | 62 | 15 | Zimmer | 11.5 | 4.1 |
| 15 | Patient 13 | Female | 55 | 15 | Zimmer | 8 | 4.1 |

The human subjects’ data were collected between 31^th^ August, 2016 to 13^th^ April, 2018and the date we conducted this study is 12^th^ June, 2018

Authors had access to information that could identify individual participants during or after data collection.

References

1. Liang Y, Yuan SS, Huan JJ, Zhang YY, Fang CY, Li JD. In Vitro Experimental Study of the Effect of Adjusting the Guide Sleeve Height and Using a Visual Direction-Indicating Guide on Implantation Accuracy. J Oral Maxillofac Surg. 2019;77:2259-2268.

2. Agerberg G. Maximal mandibular movements in young men and women. Sven Tandlak Tidskr. 1974;67: 81-100.

3. Rieder CE. Maximum mandibular opening in patients with and without a history of TMJ dysfunction. J Prosthet Dent. 1978;39: 441-446.

4. Schneider D, Marquardt P, Zwahlen M, Jung RE. A systematic review on the accuracy and the clinical outcome of computer-guided template-based implant dentistry. Clin Oral Implants Res. 2009;20 Suppl 4: 73-86.

5. Vercruyssen M, Coucke W, Naert I, Jacobs R, Teughels W, Quirynen M. Depth and lateral deviations in guided implant surgery: an RCT comparing guided surgery with mental navigation or the use of a pilot-drill template. Clin Oral Implants Res. 2015;26: 1315-1320.

6. Younes F, Cosyn J, De Bruyckere T, Cleymaet R, Bouckaert E, Eghbali A. A randomized controlled study on the accuracy of free-handed, pilot-drill guided and fully guided implant surgery in partially edentulous patients. J Clin Periodontol. 2018;45: 721-732.

7. Choi M, Romberg E, Driscoll CF. Effects of varied dimensions of surgical guides on implant angulations. J Prosthet Dent. 2004;92: 463-469.

8. Park C, Raigrodski AJ, Rosen J, Spiekerman C, London RM. Accuracy of implant placement using precision surgical guides with varying occlusogingival heights: an in vitro study. J Prosthet Dent. 2009;101: 372-381.
